# Supplementary material for: Are carbon emissions trading and green financial instruments synergistic? -Comprehensive quantitative research based on content analysis
Source: PLoS One. 2024 Mar 7;19(3):e0298601. doi: 10.1371/journal.pone.0298601 (PMC10919601; doi:10.1371/journal.pone.0298601)
Supplement: S1 Data — (ZIP) [file pone.0298601.s001.zip › Data/地级市Z_F工作报告.zip!/╣π╬≈/║╙│╪╩╨/└┤╫╘╣1⁄2╓┌║┼í╛┬φ┐╦╩2╛▌═°í┐_2018.docx]

各位代表：

现在，我代表市人民政府向大会作政府工作报告，请予审议，并请政协委员和列席会议的同志提出意见。

一、2017年工作回顾

过去一年，我们在以习近平同志为核心的党中央坚强领导下，在自治区党委、政府和市委的正确领导下，在市人大、市政协的监督支持下，全面贯彻党的十八大、十八届三中、四中、五中、六中、七中全会和党的十九大精神，坚持以习近平新时代中国特色社会主义思想为指导，按照市委“1234”总体工作思路，扎实开展“六大行动”推进年活动，全市经济呈现“总体向好、稳中提速、质量提升”的良好态势；民生持续改善，社会和谐稳定，各项事业取得新进步、迈上新台阶。初步统计，全市完成地区生产总值734.6亿元，增长7.8%；财政收入69.45亿元，增长10.5%；规模以上工业总产值403.05亿元，增长20.4%；规模以上工业增加值157.8亿元，增长9.2%；固定资产投资453.2亿元，增长12.2%；社会消费品零售总额301.2亿元，增长12.4%；城镇居民人均可支配收入25647元，增长8.4%；农村居民人均可支配收入8260元，增长10%；居民消费价格指数涨幅1.5%。多项经济指标增速进入全区前列，是我市近年来经济社会发展的最好时期。

一年来，我们主要做了以下工作：

（一）脱贫攻坚取得显著成效。市委、市政府把脱贫攻坚作为最大的政治责任、最大的民生工程和最大的发展机遇，聚焦深度贫困地区，聚焦重点领域攻坚，层层压实责任，打好脱贫攻坚战。都安县代表广西在山西太原召开的深度贫困地区脱贫攻坚座谈会上向习近平总书记作汇报。全国滇桂黔石漠化片区区域发展与扶贫攻坚现场推进会在我市召开，石漠化治理和脱贫攻坚融合发展经验获得国家层面肯定。经市级核验，实现12.6万人脱贫，145个贫困村出列，南丹县整县脱贫摘帽。

易地扶贫搬迁方面，精准确定搬迁人数，“十三五”期间全市将搬迁4.28万户18.73万人，占全区搬迁总数的25%，1009个屯纳入整屯搬迁，占全区1333个的75.7%，搬迁规模全区最大、任务最重。我们建立党政主要领导负总责、县级领导包点、市领导联系县的责任机制，突出重点，突破难点，精准施策，精准搬迁，2016年度项目投资完成率100%、竣工率99%、入住率92%；2017年度项目投资完成率83%、竣工率75%、入住率49%，综合进度保持全区前列。落实所有搬迁群众后续扶持措施，确保搬迁群众搬得出、稳得住、可发展、能致富。全区易地扶贫搬迁现场推进会连续2年在我市召开，南丹、都安、罗城、大化等县的经验和做法成为全区典范。基础设施建设方面，预脱贫村的水、电、路、房、网等基础设施全部达标，解决贫困群众安全饮水1.96万户、安全用电1431户，建设通屯道路932公里，危房改造1.02万户。公共服务保障方面，实现12.2万贫困劳动力转移就业；发放各类学生资助款3.85亿元，资助贫困学生49.35万人；选聘生态护林员9173人，管护森林面积1484万亩，兑现生态公益林补偿2.8亿元；推进农村低保制度与扶贫开发政策有效衔接，32.64万贫困群众纳入农村低保范围，重合率达82%；实施贫困人口医疗救助15.9万人次，救助金额3.55亿元。发展村级集体经济方面，2016年91个贫困村和2017年145个预脱贫村实现村集体经济项目全覆盖，年收入均实现2万元以上。财政金融扶持方面，全市共筹措财政专项扶贫资金35.24亿元，统筹整合财政涉农资金40.04亿元，新发放扶贫小额贷款3.5亿元，累计达45.5亿元；创新“保险+融资”扶贫模式，争取中国人保财险公司3亿元低息产业贷款，由政府贴息扶持种牛繁育和油茶种植，该模式入选国务院扶贫办扶贫典型案例；在全区率先为所有建档立卡贫困户69.1万人购买小额意外伤害保险；在全区率先为全市684个贫困村第一书记和139个乡镇工作队长购买人身意外伤害保险；南丹建成广西首个金融精准扶贫综合示范区。扶贫协作方面，积极拓展与深圳市在经贸、劳务、旅游、教育、人才、卫生、农业等领域的协作，利用对口帮扶资金1.83亿元，实施48个帮扶项目，帮助5247名贫困群众实现脱贫；在深圳开展多种形式农产品产销对接活动，带动3600多户贫困群众实现增收；开展各类培训32期，培训党政干部和致富带头人1606人，在深圳务工的农村劳动力达4.4万人；签订深圳·巴马大健康合作特别试验区战略合作协议；深圳市各界捐款捐物3000多万元。社会扶贫方面，广泛开展“千企助百村”活动，新奥集团捐赠1200万元帮扶贫困学子，一批有爱心的企业和社会人士无私捐助9.6亿元，形成了全社会关心扶贫、参与扶贫、支持扶贫的浓厚氛围。

（二）“三农”工作和“十大百万”扶贫产业工程取得新进展。全市粮食总产量实现106万吨。创建各级现代特色农业示范区206个，其中建成自治区级15个；建成现代林业示范区5个，新建林下经济示范基地11个。新型农业经营主体不断壮大，新增农民合作社781家，达4665家，其中684个贫困村实现全覆盖；新增家庭农场113家，达662家；新认定市级农业产业化龙头企业5家，累计达57家。新认定“三品一标”产品41个、基地面积117万亩。农业基础设施建设加强，新增恢复和改善灌溉面积15.5万亩。完成11个县（区）全域永久基本农田划定工作，连续18年实现耕地占补平衡。

市委、市政府将产业发展作为脱贫攻坚的关键，精心谋划、大力实施“十大百万”扶贫产业工程，确保群众有稳定的、可持续的收入来源。通过规划引领、示范带动、品牌打造，扶贫产业工程取得良好成效。全市新种核桃14万亩，总面积达255万亩，建成“万千百”高产栽培核桃示范基地1185个30万亩；新种油茶12万亩，总面积突破100万亩，建设千亩油茶高产示范基地14个；分别创建7个种牛、10个种羊繁育示范基地，肉牛、肉羊饲养量及出栏量居全区第一；内陆大水面开发面积80万亩，居全区第一；新种桑园面积3万亩，总面积达85万亩，桑蚕生产规模稳居全国第一；“三特”水果、糖料蔗、香猪、长寿生态富硒农产品等产业完成年度目标任务。都安种牛繁育，罗城高产油茶种植，天峨“三特”水果种植，凤山、金城江和东兰核桃管护成为全市示范。

（三）工业转型升级步伐加快。围绕“做大总量，做长链条，做优结构”目标，出台培育壮大“五大百亿元产业”实施方案、市级工业园区转型升级提质发展行动计划等措施，推动主要行业保持较快增长，有色金属、建材、茧丝绸等产业产值分别增长43%、40%、18%。南方公司销售收入突破百亿元，实现税收7.5亿元（含关税），成为河池首家百亿元企业。加快推进园区建设，全市工业园区完成工业总产值260亿元，增加值58亿元。大任产业园基础设施建设累计完成投资31.5亿元，园区建设取得突破性进展；鑫锋、生富、金兴、华远等中心城区周边企业搬迁入园，累计完成投资19亿元；引进誉升、新奥生富、明鑫环保、广投中燃等企业入园投资建厂。加快推进重点项目，南方公司锑银多金属综合回收项目竣工投产；鑫锋公司与全国最大的蓄电池企业超威集团合作的蓄电池项目投产，年产值30亿元以上；琦泉生物质发电、嘉欣丝绸园等一批项目开工建设。积极培育新的增长点，推动特色酒、优质饮用水、绿色长寿食品等产业规模化、品牌化、高端化发展，丹泉、天龙泉成为全区酿酒龙头企业，巴马丽琅、九千万、木论思泉等品牌优质饮用水畅销区内外，食品加工、优质饮用水产业产值分别增长12%、23%；成功举办首届河池（巴马）国际长寿养生健康饮用水博览会。大力实施创新驱动战略，鼓励引导企业加大创新力度，新增高新技术企业2家，引进浪潮集团建成河池云计算创新中心和“双创”基地；强化产学研合作，促进科技成果转化14项；发明专利申请量1454件；创建自治区级农业科技园区5家。质量强市战略成效明显，质量工作代表广西接受国务院考核获得充分肯定；颁发“第二届市长质量奖”；9个品牌荣获“广西名牌”称号；核准注册“长寿巴马”商标，涵盖商标分类全部45个类别，巴马县成为全国第一个将县域公共品牌成功进行商标全类别注册的县。

（四）现代服务业加快发展。坚持因地制宜，特色发展，推动服务业成为河池经济转型发展的重要动力，服务业对经济增长贡献率超过50%。全域旅游加快发展，扎实推进巴马长寿养生国际旅游区建设，实施基础设施建设新三年行动计划项目27个，完成投资3.9亿元。推进巴马百魔洞国际养生度假区、都安三岛湾国际度假区等重大旅游项目，完成投资6.7亿元。建成赐福湖国际长寿养生度假小镇（一期）。引进新奥集团，全面启动红水河健康之旅项目，计划投资100亿元打造风情小镇、地质公园、豪华游轮等高端旅游产品，是我市继巴马国际旅游区建设之后的又一重大旅游项目，将推动河池旅游产业提质发展。实施宜州刘三姐故乡风情旅游区、环江木论喀斯特生态旅游景区等百里龙江民族文化生态旅游带项目24个，完成投资10.3亿元。南丹全域旅游异军突起，开工建设“千家瑶寨·万户瑶乡”旅游综合体、丹泉小镇、丹炉山风景区、温泉公园等一批旅游项目。巴马入选第二批国家级旅游业改革创新先行区，巴马甲篆镇入选首批“广西养生养老小镇” ，凤山入围广西特色旅游名县创建县、荣获“全国森林康养基地”称号。宜州区下枧河获评首届全国10条“最美家乡河”之一。组建巴马国际旅游区投资发展集团公司、河池市旅游发展投资公司。旅游业保持较快发展势头，全市接待游客2632万人次，增长22%；旅游总消费297亿元，增长27%。积极发展商贸物流业，高德物流仓储基地、惠元汽车配件城建成投入使用，都安冷链仓储交易市场等项目竣工，总投资30亿元的红星美凯龙商业广场项目开工建设。新增4个县获批全国电子商务进农村综合示范县。举办系列农产品产销对接活动，交易额超亿元。抓好财税金融工作，发挥财政职能作用，积极筹措资金，支持实体经济发展，保障民生支出，实现财政平稳有序运行。全市新增存款126亿元，存款余额1127亿元；新增贷款86亿元，贷款余额661亿元；实现保费收入20亿元，增长17%；企业上市挂牌工作稳步推进，14家企业进入自治区企业上市后备资源库；组建河池市小微企业融资担保公司，融资能力不断提升；深化农村金融改革，全市设立“两点一站”2076个，助农取款在农村实现全覆盖。

（五）基础设施建设取得新突破。建立完善项目谋划和促进项目建设的新机制，为培育新的经济增长点、做大经济总量和改善基础设施条件夯实基础。加大项目谋划力度，围绕基础设施建设、产业发展、产业转型升级等重点领域，确保项目前期经费，谋划项目699个、总投资2400亿元。深入实施交通基础设施建设大会战，完成投资90亿元。公路建设方面，河池至百色高速公路加快推进，新开工贺州至巴马（都安至巴马段）、融水至河池（河池段）、乐业至百色（天峨段）3条高速公路；建成环江至东江、大任产业园入园公路等二级路网项目178公里；新开工建设凤山至乐业二级公路；改扩建农村公路322公里。加快推进南丹至天峨下老高速公路、环江至河池一级公路、凤山至巴马一级公路、东兰至都安大兴二级公路等项目前期工作。铁路建设方面，贵阳至南宁高速铁路（河池段）征地拆迁全面开展，实现实质性开工；柳州至河池（金城江）城际铁路前期工作加快推进。水运建设方面，龙滩升船机工程按过船能力1000吨的标准启动方案调整论证，加快推进大化船闸和岩滩升船机改扩建项目前期工作。深入实施水利基础设施建设大会战，凤山社更水库、罗城大山水库等重点水源、农村水利、防洪减灾三大工程116个项目顺利实施，完成投资12亿元；完成桂北贫困地区（河池）特色农业节水灌溉项目、桂西北扶贫治旱红水河灌区工程规划前期工作。统筹推进重大项目建设，自治区级市级层面项目完成投资265亿元；2015年和2016年中央预算内投资项目超额完成投资任务；2017年中央预算内投资项目开工建设154项，完成投资18.4亿元。电网建设投资完成10.2亿元。

（六）统筹城乡协调发展。城乡规划进一步健全，稳妥推进行政区划调整，完成宜州撤市设区，宜州新区规划有序开展。完成河池市土地利用总体规划、城市总体规划修编，初步完成宜州新区（一期）、东江高铁新区、金城江城区等控制性详细规划编制。抓好中心城区重点项目建设，河池市老年大学、河池政协文史馆、翠竹路、星河路、动物园路、老年门球场等一批项目竣工或投入使用，市体育场、市文化宫等项目加快推进。实施大县城战略，加快推进宜州、环江、东兰等自治区级新型城镇化示范县项目。村镇建设有序推进，23个百镇建设示范、54个全区乡土特色示范点项目顺利实施，宜州刘三姐镇入选全国特色小镇。市容市貌持续改善，深入开展环境卫生专项整治，严厉打击“两违”行为，营造干净、整洁、有序的人居环境。南丹县获评“国家园林县城”称号。安居工程建设扎实推进，老地委、人民厂、老虎山片区棚户区改造项目加快建设；全市保障性安居工程和棚户区改造新开工6059套，分配入住2292套。

（七）生态文明建设取得新进步。加大生态保护力度，大力开展石漠化治理，治理岩溶380平方公里、治理石漠化150平方公里；完成植树造林32万亩，森林覆盖率达68.83%。加大环境问题整治力度，实施土壤污染防治先行区、龙岩滩水库生态环境保护、刁江流域重金属污染综合治理等环保项目77个，获得上级资金支持4亿元。全力抓好中央环保督察反馈的12个问题的整改工作，已完成整改7个，正在抓紧整改5个。全面建立“河长制”，抓好水污染防治，32个重要水功能区水质达标率100%。开展大气污染防治，金城江城区环境空气优良率93%，10个县（区）城区空气环境质量持续稳定达到国家二级标准。地质灾害防治不断加强，投入5514万元，治理地质灾害隐患点65个。推进“美丽河池·宜居乡村”建设，开展产业富民、服务惠民、基础便民专项行动，创建产业富民示范点8个，建设760个行政村综合服务中心，完成农村改厨、改厕26.9万户，改圈3.2万户，农村人居环境质量明显提升。

（八）改革开放持续深化。坚持以供给侧结构性改革为主线，推动改革举措落地见效。全面落实“三去一降一补”政策，化解煤炭过剩产能165万吨，8家有色金属企业拆除落后工艺设备；全面落实自治区降成本41条、28条措施，减免税款15亿元，争取电力直接交易、降低高速公路收费、降低金城江区和宜州区城镇土地使用税等政策，为企业减负5亿元。推进“放管服”改革，提前完成市县政府部门权责清单“两单融合”工作；取消行政审批事项54 项，调整行政审批事项268项，市级行政审批时限提速60%。积极推进国资国企改革，完成金河公司破产重整，妥善处置河化集团改制遗留问题，完成4家企业政企分开。推进商事制度改革，全市发放“多证合一、一照一码”营业执照1373本，注册资金60多亿元。加快推进国有林场改革，完成环江、南丹国有林场试点改革任务，其他9个县（区）主体改革任务按时完成，5296名职工纳入社保范围。积极推进医药卫生体制改革，建立医疗联合体23个，10个县（区）通过自治区公立医院综合改革效果评价核验。统筹推进教育、科技、财税、供销社、安全生产、公证体制等领域改革。加大对外开放力度，积极融入“一带一路”建设，成功举办巴马论坛——第二届中国—东盟传统医药健康旅游国际论坛，加强与“珠三角”“长三角”等发达地区和周边地区的合作交流，先后与深圳、柳州、百色以及贵州黔南州等地达成合作协议，推进产业、基础设施、文化旅游联动发展。加大招商引资力度，到位内外资331亿元，其中全口径外资到位1.45亿美元。签订重点项目合作协议98个，总投资907亿元。

（九）民生持续改善。继续实施为民办实事工程，集中力量做好普惠性、基础性、兜底性民生建设，人民群众的获得感、幸福感、安全感全面提升。教育事业不断发展，新建公办幼儿园10所，实施中小学、普通高中基础设施建设项目493个，办学条件进一步改善。学前三年教育毛入园率达82%，义务教育巩固率达93%，高中阶段教育毛入学率达85.5%，金城江区通过国家义务教育均衡发展督导评估认定，环江、南丹、天峨、东兰、凤山、大化6县通过自治区义务教育均衡发展督导评估认定。积极发展特殊教育，适龄残疾儿童入学率达90%。组建河池市职业教育集团，职业教育发展迈出新步伐。就业创业工作有序推进，落实创业优惠政策，鼓励大众创业万众创新，全市城镇新增就业1.74万人，新增农村劳动力转移就业6.98万人次。社会保障水平不断提高，在全区率先实施城镇职工医疗“双保险”，惠及25万参保职工。顺利完成新农合与城镇医疗保险整合工作，实现城乡居民基本医疗保险管理制度“八统一”，城乡居民养老保险参保率99.6%、养老金发放率100%。城乡低保保障标准进一步提高。健康河池建设加快推进，金城江城区顺利通过自治区卫生城市复审，天峨、巴马、凤山获得自治区卫生县城荣誉称号。基本公共卫生服务、重大传染病防控、中医药民族医药等卫生事业全面发展。继续稳妥实施全面二孩政策，全市生育水平可控，人口性别结构更趋合理。公共文化服务体系日益健全，社会主义核心价值观深入人心，精神文明创建活动成效显著，6个单位荣获全国文明单位，2所学校荣获全国文明校园。新建村级公共服务中心124个。城乡群众性文体活动丰富多彩，成功举办第十八届铜鼓山歌艺术节。全民健身活动蓬勃开展，竞技体育成绩喜人。成功举办全国大学生五人制足球联赛、全国山地自行车冠军赛（南丹站）等国家级赛事。加强和创新社会治理，完善社会治安防控体系建设，探索建立社会应急联动机制成为全区示范。成功破获“2017—189”特大制毒案件和“6·9”虚开发票案，得到公安部肯定。加强平安河池建设，我市荣获全区平安建设先进市、天峨县荣获全国平安建设先进县、凤山县荣获全国信访“三无”县，市公安局金城江分局荣获“全国优秀公安局”。深入开展“矛盾纠纷化解攻坚年”活动，推行“家门口”信访、乡村巡回接访、第三方参与信访等模式，把矛盾纠纷化解在基层。水库库区移民遗留问题处置取得良好成效。全面落实安全生产责任，安全生产形势保持稳定。加强食品药品安全监管，食品药品安全社会满意度持续提升。国防动员和后备力量建设不断加强，双拥共建力度加大。认真贯彻落实党的民族政策，成功举办环江毛南族自治县成立30周年县庆活动，挖掘和弘扬民族文化，举办系列少数民族节庆活动。完成新一届村（社区）换届选举工作。

（十）政府自身建设不断加强。以高度的政治自觉和政治责任做好迎接学习宣传贯彻党的十九大各项工作，深入学习贯彻习近平总书记系列重要讲话和视察广西重要讲话精神，推动“两学一做”学习教育常态化制度化。牢固树立“四个意识”，坚定“四个自信”，在政治立场、政治方向、政治原则、政治道路上同以习近平同志为核心的党中央保持高度一致，确保中央政令畅通。认真贯彻落实自治区党委、政府和市委的各项决策部署，认真执行市人大及其常委会的决定决议，自觉接受市人大及其常委会的法律监督、工作监督和市政协的民主监督。把政府活动全面纳入法治轨道，重大行政决策合法性审查、行政首长出庭应诉、行政复议规范化等工作成效显著。深化政务公开，网络问政制度化、规范化、常态化运行。扎实改进作风，带头执行中央八项规定，密切联系群众。严格落实全面从严治党责任，切实抓好意识形态工作，扎实推进党风廉政建设和反腐败工作，深入查处发生在群众身边的“四风”和腐败问题，严肃查处了一批违纪案件，群众对反腐败成效满意度进一步提升。积极推进审计全覆盖，扶贫领域和自然资源审计取得新成效。

统计、司法行政、税务、外事、宗教、侨务、机关事务、消防、保密、检验检疫、档案、地方志、通信、海事、气象、地震、水文等工作迈出新步伐。老干部、妇女儿童、老龄、残疾人保障等事业取得新进展。工会、共青团、妇联等群团组织在经济社会发展中发挥了重要作用。驻河池中直、区直各单位支持地方发展和自身业绩取得新成效。

各位代表！刚刚过去的2017年，我们凝心聚力、砥砺奋进，顶住了压力，经受住了考验，发展质量和效益不断提升，为打赢脱贫攻坚战、全面建成小康社会奠定了坚实基础。这是市委统揽全局、科学谋划的结果，是市人大依法监督、市政协民主监督、鼎力支持的结果，是全市广大干部群众团结一心、合力攻坚的结果，是离退休老干部鼓励支持、建言献策的结果，是驻河池人民解放军、武警部队和社会各界广泛参与、大力支持的结果。在此，我谨代表市人民政府，向全市各族人民，向人大代表、政协委员，向各民主党派、工商联、无党派人士、各人民团体，向驻河池人民解放军、武警部队官兵，向所有关心支持河池发展的社会各界人士，表示崇高的敬意和衷心的感谢！

在看到成绩的同时，我们也清醒地认识到，受历史及自然地理条件等因素制约，河池仍然是一个欠发达后发展地区，还面临着一些困难和问题：经济基础薄弱，经济结构不优，创新动能不足；贫困人口多、贫困面大、贫困程度深，脱贫攻坚任务依然繁重艰巨；实体经济仍面临不少困难，企业融资压力大，民间投资活力不足，科技含量高、带动能力强的好项目大项目仍然不多；历史遗留环境问题整治还需要进一步加大力度；少数干部责任担当和创新意识不强，不作为、慢作为、不会为现象依然存在，政府效能、服务水平还需进一步提高。我们将坚持问题导向，采取有力措施，努力把工作做得更好，决不辜负全市人民的期望和重托。

二、2018年工作安排

今年是贯彻落实党的十九大精神的开局之年，是改革开放40周年，是决胜全面建成小康社会、实施“十三五”规划承上启下的关键一年。党的十九大作出了中国特色社会主义进入了新时代的重大政治论断，开启了坚持和发展中国特色社会主义的新纪元。“全面深入学习贯彻党的十九大精神，用习近平新时代中国特色社会主义思想武装头脑、指导实践、推动工作，大力弘扬河池精神，深入开展‘六大行动’，决战贫困决胜小康，齐心奋力谱写新时代河池新篇章”，这是新时代河池发展的主题。我们要充分利用国家实施“一带一路”建设、西部大开发、脱贫攻坚、左右江革命老区振兴规划、滇桂黔石漠化治理等重大战略和自治区履行“三大定位”使命、推进富民兴桂等决策部署的重大机遇，着力解决好发展不平衡不充分问题，大力提升发展质量和效益，更好满足人民日益增长的美好生活需要。

进入新时代，贯彻新思想，追求新目标，今年政府工作的总体要求是：全面贯彻党的十九大精神，以习近平新时代中国特色社会主义思想为指导，坚持稳中求进工作总基调，坚持新发展理念，按照高质量发展要求，统筹推进“五位一体”总体布局和协调推进“四个全面”战略布局，紧紧围绕自治区第十一次党代会提出的营造“三大生态”、实现“两个建成”战略部署和市第四次党代会提出的“1234”总体工作思路，坚持以供给侧结构性改革为主线，以开展“六大行动”提质年活动为抓手，以全面深化改革为动力，以脱贫攻坚为重点，狠抓产业发展和项目建设，统筹推进稳增长、促改革、调结构、惠民生、防风险各项工作，确保全市经济持续健康发展、社会和谐稳定。

全市经济社会发展的预期目标是：地区生产总值增长7%，规模以上工业总产值增长18%，规模以上工业增加值增长9%，固定资产投资增长15%，财政收入增长8%（奋斗目标10%），社会消费品零售总额增长11%，全体居民人均可支配收入增长8%，居民消费价格指数控制在3.5%以内，城镇登记失业率控制3%以内，常住人口城镇化率39.5%，节能减排降碳控制在自治区下达目标内。

围绕实现上述目标，重点做好十个方面的工作：

（一）全力以赴抓好脱贫攻坚，坚决完成脱贫摘帽任务。让贫困人口和贫困地区同全国一道进入全面小康社会是我们党的庄严承诺。坚持把脱贫攻坚作为当前最大的政治任务、最大的民生工程、最大的发展机遇来抓，坚持精准扶贫、精准脱贫，全面完成今年贫困村、贫困人口脱贫摘帽任务，确保金城江区、天峨县脱贫摘帽，力争环江县脱贫摘帽，做到脱真贫、真脱贫。

进一步压实责任。强化党政一把手负总责的责任制，层层压实脱贫攻坚责任。进一步充实市、县脱贫攻坚指挥部力量。各县（区）主要领导要履行脱贫攻坚第一责任人责任，集中精力抓落实，做到亲自挂帅、亲自部署、亲自督办，及时研究解决脱贫攻坚重大问题。市直各单位主要领导要履行帮扶单位第一责任人责任，督促本单位干部职工抓好后援帮扶和结对帮扶工作。强化督促检查，组建专业督查队伍，选派有责任心、原则性强、熟悉业务、熟悉政策的干部，专职抓督查工作；通过跟踪督查、随机抽查、明察暗访等方式，采取管用、有力的办法，做到奖罚分明，推动脱贫攻坚各项工作落实。

继续抓好产业扶贫。围绕“十大百万”扶贫产业工程，按照“长中短”相结合，因户施策，实现扶贫产业对贫困户全覆盖，确保每个贫困户发展1项以上增收产业并实现增收1000元以上。建立健全企业与贫困农户的利益联结机制，完善“公司+基地（合作社）+贫困户”模式，每个贫困村合作社发展1项以上覆盖面广的脱贫产业。

抓好易地扶贫搬迁。全面启动后三年易地扶贫搬迁项目建设；其中2018年度项目要全部竣工，3.38万贫困群众全部搬迁入住。每个项目安置点继续落实1名县级领导，实行“八包”责任制，以住房、基础设施、公共服务配套建设和产业就业扶持为重点，提高住房竣工率、投资完成率、搬迁入住率、移民脱贫率，确保竣工一批、交房一批、入住一批、脱贫一批，并顺利通过国家考核验收。

抓好基础设施建设。严格按照国家“两不愁、三保障”和自治区关于贫困户“八有一超”、贫困村“十一有一低于”、贫困县“九有一低于”的脱贫摘帽验收标准，补齐基础设施建设短板。对稳固住房、20户以上的屯级道路等水、电、路、房、网项目做到早计划、早安排，确保所有项目提前开工建设，及早竣工投入使用。

提升公共服务水平。抓好转移就业工作，帮扶引导贫困群众外出务工；落实生态补偿政策，按时兑现2.8亿元生态补偿资金，发放护林员管护补助5700万元；全面落实教育精准扶贫扶持政策，不让一名学生因贫失学；做好医疗救助工作，对因病致贫、因病返贫的贫困人口，纳入重特大疾病救助范围；进一步完善兜底措施，做到应保尽保、托住底线。

发展壮大村级集体经济。因村精准施策，通过盘活资源、发展产业、帮扶合作、资金扶持、监督管理等措施，发展资源型、产业型、股份合作型、旅游型、服务型、租赁型等多种模式的村级集体经济，确保每个贫困村有1-2个覆盖60%以上贫困户的产业，确保每个贫困村集体经济年收入到2020年达到5万元以上。

深化扶贫协作。强化深圳·河池在旅游扶贫、劳务合作、土地增减挂钩、人才交流、农产品开发推销、招商引资、社会参与等方面的协作，抓好扶贫协作项目实施。同时，坚持专项扶贫、行业扶贫和社会扶贫联动，深入推进“千企助百村”活动，积极引导社会力量参与帮扶，形成全社会共同参与扶贫的强大合力。

激发贫困群众的内生动力。注重扶贫与扶志、扶智相结合，引导贫困群众树立主体意识，发扬自力更生精神，激发改变贫困面貌的干劲和决心，变“要我脱贫”为“我要脱贫”，靠自己的努力改变命运，巩固扶贫成果，提高脱贫质量。

（二）实施乡村振兴战略，推动“三农”工作实现新发展。坚持农业农村优先发展，全面实施乡村振兴战略，按照产业兴旺、生态宜居、乡风文明、治理有效、生活富裕的要求，加快推进农业农村现代化。

突出抓好“十大百万”扶贫产业工程。加快培育形成有规模、有特色、有品牌、有效益、能够辐射带动群众稳定增收的主导产业。

——扩大产业规模。坚持新种、改造、管护并重，进一步扩大产业发展规模。新种补种核桃17万亩，采取有力措施强化核桃管护，重点管护30万亩“百千万”核桃示范基地；新种“三特”水果7万亩，总面积达98万亩；新种油茶15万亩，低产改造10万亩，总面积达120万亩；新增糖料蔗种植11万亩，总面积达95万亩，建设“双高”基地4万亩；新增桑园5万亩，总面积达90万亩；实施板栗低产林改造5万亩；新增肉牛、肉羊出栏量9万头（只），出栏总量达133万头（只）；新增香猪（含黑土猪）15万头，出栏总量达170万头；新增淡水生态养殖6万亩，达86万亩；新增长寿生态富硒农产品基地10万亩，总面积达80万亩。

——创建示范基地。每个产业创建1个以上可借鉴、可复制、可辐射带动60%以上贫困户增收的示范基地。在金城江区、凤山县打造万亩核桃示范基地；在罗城县打造万亩油茶示范基地、在东兰县打造油茶低产改造示范基地和板栗示范基地；在宜州区打造万亩糖料蔗“双高”示范基地和万亩桑蚕产业示范基地；在天峨县、环江县、罗城县各创建1个精品“三特”水果示范基地；在南丹县打造长寿生态富硒产业示范基地；在都安县打造肉牛、肉羊示范养殖基地；在大化县打造淡水生态示范养殖基地；在巴马县打造香猪（含黑土猪）示范养殖基地。

——打造河池农产品品牌。按照“六统一”的要求，打响“绿色长寿养生”农产品品牌。重点抓好“三品一标”建设，制定每个产业标准化生产方案，新增认证“三品一标”产品15个以上。打造1—2个公共品牌，并注册登记商标，提高知名度和美誉度。

——抓好农产品加工销售。充分利用深圳帮扶和电商平台，做好线上和线下农产品的宣传和营销服务工作。抓好农产品精深加工，延伸产业链，提升附加值，推动一二三产业融合发展。加大农产品推介展销力度，面向全国、全区开展名特优农产品展销活动。

——加大资金扶持力度。进一步整合各类涉农资金投入。实施特色农业保险试点，减少贫困群众养殖、种植风险。加大对油茶、肉牛、肉羊等重点产业的贷款资金扶持力度。坚持“户贷户用户还”原则，做好扶贫小额信贷工作。

推动现代特色农业示范区建设。抓好粮食生产，确保粮食安全。紧紧围绕示范区“增点、扩面、提质、升级”的目标，不断增加示范区的数量，进一步扩大示范区区域、品种、产业、行业覆盖面，进一步提高示范区发展质量和效益。每个县（区）建成1个以上自治区级示范区、2个以上县级示范区，县乡两级示范区（园）数占所在县（区）乡镇总数的30%以上，每个行政村至少有1个示范点。在宜州区打造桑蚕产业生产机械化创新示范基地，在天峨县打造山区桃李产业生产机械化创新示范基地。

培育新型经营主体。培育有文化、懂技术、会经营的新型农民，培养造就一支懂农业、爱农村、爱农民的“三农”队伍。新增农民合作社250个、家庭农场110家，市级以上农业产业化龙头企业保持在60家以上。表彰奖励一批新型农业经营主体，营造产业发展的良好氛围。

深化农村改革。全面完成农村承包土地经营权颁证工作，继续保持土地承包关系稳定，在第二轮土地承包到期后再延长三十年。加快南丹县农村产权流转交易市场建设，探索农村产权抵押贷款模式。

（三）加快产业转型升级，做大工业实体经济。坚持质量第一、效益优先，在推动产业优化升级、转变发展方式、提高创新能力上下功夫，推动经济持续健康发展。

提升园区发展水平。抓好园区基础设施建设、特色产业发展和招商引资，优化园区服务，做大园区经济总量，促进园区扩能增效。市级工业园区实现总产值252亿元，增长20%；完成增加值60亿元，增长20%。每个园区围绕重点产业策划10个以上项目，力争引进重大项目10个以上，其中亿元以上项目5个以上。突出抓好大任产业园建设，进一步完善路网、供水、供电、供气、通讯等基础设施建设，推进园区至拉浪公路、标准厂房等建设，做好土地规划修编及收储工作；重点抓好园区招商引资，引进亿元以上项目10个以上，到位资金30亿元以上；加快项目建设进度，推动8个项目竣工投产，形成新的工业增量；园区完成工业投资10亿元以上，实现产值30亿元以上。

抓好重大工业项目建设。新开工南方公司年产3万吨锡冶炼、超威鑫锋公司新型蓄电池技改（二期）、誉升公司年产10万吨电锌、鑫锋公司年处理20万吨废铅酸蓄电池等项目；续建金兴公司技改、金鹏公司综合回收利用工程、嘉欣丝绸园、宜州琦泉生物质发电等31个项目；竣工投产南方公司20万吨次氧化锌、生富公司年产1.5万吨锑金属技改、华远公司年产1.5万吨锑品深加工技改、五和博澳公司桑树资源综合开发等项目。通过重大项目建设，带动有色金属、茧丝绸、电力等传统产业提质发展，产值分别达到169亿元、37亿元、120亿元，分别增长30%、25%、15%。

培育新的经济增长点。积极引导酿酒、饮用水重点企业强化营销，开拓市场，打响品牌，进一步释放产能。重点推进天龙泉公司年产3万吨黄酒营养酒配套设施、中源山泉公司年产50万吨山泉水技改等项目建设；酿酒、饮用水产业实现产值60亿元以上。实施一批碳酸钙重大项目，抓好凤山华泰公司碳酸钙技改、南丹联丰公司大理石板材加工等项目，实现产值10亿元以上。

加大服务企业力度。全面落实自治区降成本41条和28条措施，进一步降低实体经济企业成本。进一步扩大电力直接交易规模，降低企业用电成本。深入开展服务企业大行动，加大对南方、丹泉、天龙泉、超威鑫锋、生富、华锡等53家产值超亿元重点企业的扶持服务力度。继续争取国家和自治区支持，推进矿产资源配置工作，努力实现矿产资源“三就地”。设立工业产业发展基金，吸引社会资金推动产业转型升级。加大对企业的融资服务力度，开展企业“惠企贷”融资服务。实施企业上规培育计划，培育规模以上企业20家以上。全面开展质量提升行动，推进生态原产地产品保护，培育广西名牌产品5个以上，创建国家级品牌。加大科技创新服务力度，培育高新技术企业7家以上，推动宜州茧丝绸产业孵化器、科技创客城等自治区级创新创业平台建设，全市创业孵化企业达50家以上。

（四）推进现代服务业提质增效，培育发展新动能。以旅游业为重点，带动现代服务业发展提速、比重提高、质量提升。

加快发展全域旅游。以建设巴马国际旅游区、红水河健康养生之旅、百里龙江民族文化生态旅游带、南丹全域旅游为重点，推动全市旅游业提质增效。全年接待国内外旅游者增长16%以上，旅游总消费增长23%以上，旅游投资增长30%以上。加快巴马国际旅游区建设，力争年内建成几个特色鲜明、带动性强、社会影响大的精品旅游项目。继续实施基础设施建设三年行动计划，加快推进赐福湖小镇（二期），巴马命河-水晶宫、东兰红水河第一湾、凤山三门海等景区基础设施项目35个，完成投资10亿元以上。规划建设深圳·巴马大健康合作特别试验区。谋划巴马至百色巴马机场的高速公路。加快推进红水河健康养生之旅项目建设，抓好大化瑶泉生态旅游小镇，天峨龙滩大峡谷景区、川洞河燕子湖景区、蓝衣壮小镇，东兰河谷小镇等项目建设；开通旅游航线，完成投资10亿元以上。加快建设百里龙江民族文化生态旅游带，重点推进金城江姆洛甲旅游区、宜州刘三姐故乡风情旅游区、罗城棉花天坑旅游度假区、环江木论喀斯特生态旅游等项目建设；抓好南丹“千家瑶寨·万户瑶乡”旅游综合体、丹泉小镇、温泉养生度假区（二期）、丹炉山等景区景点建设，完成投资21亿元。完善旅游公共配套设施建设，推进“厕所革命”，新建、改建金城江至巴马二级公路沿线及中心城区厕所；全市新建、改建旅游厕所75座。建设11个汽车旅游营地。完善旅游咨询服务体系、旅游标识系统等公共配套设施建设。加大品牌宣传力度，打造长寿、生态、民族、红色品牌，做好首个世界长寿市品牌宣传，让世界了解河池、了解巴马；抓好旅游精品线路的宣传推广，提高河池旅游的知名度和影响力，吸引更多的游客来河池观光旅游。

积极发展健康产业。充分发挥“巴马论坛”影响力，组建水、地磁等长寿因素研究所，力争月月开展主题活动。开展养生养老小镇创建工作，推动巴马县巴马镇、凤山县三门海镇、东兰县武篆镇、金城江区六甲镇、罗城县小长安镇争创全区健康养生养老小镇。积极创建国家中医药健康旅游示范基地，继续打造桂西北养生养老长寿产业示范区。

大力发展商贸物流业。加快红星美凯龙商业广场、市粮食产业物流园、宜州东盟国际茧丝绸交易中心等一批商贸物流项目建设。实施乡镇农贸市场升级改造工程，重点改造12个乡镇农贸市场。抓好全国电子商务进农村综合示范县项目建设，推进市县乡村四级电商服务网络建设。引导企业推行“线上推广交易+线下体验服务”新模式，带动商贸物流业上档次、上水平。

协调发展各类服务业。培育壮大信息科技、健康养生、会展营销等现代新兴服务业，推进交通运输、住宿餐饮、家政服务等传统产业提质升级。巩固房地产市场向好态势，建立能够满足多层次需求的住房供应体系。积极培育民族服饰、民族表演等特色文化产业，扶持壮大一批文化企业。

（五）加快重大项目建设，夯实发展后劲。坚持把投资和项目建设作为培育新的经济增长点、做大经济总量、改善基础设施条件的重点工作来抓，为经济持续健康发展提供有力支撑。

加大项目谋划储备力度。聚焦“一带一路”、“中新南向通道”、珠江—西江经济带、左右江革命老区发展振兴规划、柳来河一体化等国家和自治区的发展战略，确保前期工作经费，积极借助专业机构，围绕脱贫攻坚、工业转型升级、特色产业发展、生态养生旅游、交通水利基础设施等领域，精心谋划一批重大项目。全年策划重大项目800个以上，其中5000万元以上项目190个以上，500-5000万元项目610个以上。

统筹推进重大项目建设。加快自治区和市级层面统筹推进的400项重大项目建设，确保完成投资340亿元以上。狠抓中央预算内投资项目建设，2017年项目要全部开工建设，投资完成率90%以上；2018年项目开工率90%以上，投资完成率60%以上。

深入实施交通基础设施建设大会战。完成投资110亿元以上。公路方面，以实现县县通高速，打通往南宁、桂林和贵州的便捷通道为目标，抓好交通项目建设。建成河池至百色高速公路；加快推进贺州至巴马（都安至巴马段）、融水至河池（河池段）、乐业至百色（天峨段）3条高速公路建设；优化南丹至天峨下老高速公路建设方案，力争开工建设；积极谋划宜州至忻城，荔波经环江至德胜，天峨经凤山、巴马至田东、大新高速公路项目。抓好一、二级公路重点路网项目建设，新开工环江至河池一级公路、凤山至巴马一级公路及东兰至都安大兴、罗城四把至环江二级公路等重点路网项目；续建宜州经北牙至龙头、南丹吾隘至东兰、贵州瑶山至南丹、天峨至凤山（天峨段）、三门海至乐业（凤山段）、大化绕城线等二级公路项目。启动乡乡通二级（三级）公路建设工程。实施一批乡村路网项目，“建好、管好、护好、运营好”农村公路。铁路方面，加快贵阳至南宁高速铁路（河池段）建设，力争开工建设柳州至河池（金城江）城际铁路，积极做好河池经东兰、巴马至百色城际铁路项目前期工作。水运方面，完成岩滩升船机改造、大化船闸改扩建的前期工作，争取项目立项；按过船能力1000吨的标准推进龙滩升船机工程建设。

深入实施水利基础设施建设大会战。完成投资12亿元以上。全面实施重点水源、农村水利、防洪减灾三大工程，完成金城江花任水库，罗城白坝水库，南丹火幕水库，东兰香河水库，凤山乔音水库扩容工程、社更水库6个项目建设任务，开工建设2座中型水库，加固9座病险水库。建成小型农田水利项目29处，新增和恢复农田灌溉面积12万亩，改善灌溉面积6万亩；建设河流河堤及护岸工程40公里，提升抗旱减灾能力。抓好桂北贫困地区（河池）特色农业高效节水灌溉项目建设，做好桂西北扶贫治旱红水河灌区工程项目的争取工作，完成罗城县板阳东、环江县上帮、天峨县索法、南丹县天生桥水库扩容、凤山县上林、都安县板岭6座水库前期工作。

抓好能源信息基础设施建设。大力实施县县通天然气工程，推进天然气支线管道及县域支线同步配套建设。开工建设金城江重点水环境治理及水电站工程。加快罗城高帮山风电场、环江界子良风电场和驯乐风电场等项目建设。继续抓好农村电网升级改造，完成电网建设投资8亿元。开工新建一批移动4G网络基站和小区宽带项目。全面实施大数据发展行动，充分利用浪潮集团云计算中心平台，推动智慧城市建设，完善智慧政务、智慧产业、智慧民生等大数据系统，实现资源共享。

（六）加快新型城镇化进程，打造宜居城市。以创建宜居城市为载体，加快补齐城镇短板，提升综合承载力和辐射功能，让人民群众生活得更方便、更舒心、更美好。

加快推进宜州新区建设。按照“三年打基础，五年出成效，十年现新城”的要求，把新区建设成为自然生态新都市、绿色产业新高地、经济增长新一极。全面完成河池市城市总体规划修编、金城江—宜州城市走廊规划和各专项规划的编制，逐步构建“一市两区”的城市空间架构。加快建设广西园林园艺博览会（河池）项目；重点推进金山大道、开元大道、新城大道等“四纵三横”城市干道建设；开工建设金山湖小区；扎实开展科教园、市政服务片区、洛寿产业园、物流园等项目前期工作。

推进中心城区重点项目建设。有序启动市行政中心驻地迁移工作。实施金城江至宜州一级公路改造。启动东江高铁新区规划建设。开工建设金城江城区道路“白改黑”、城市亮化工程、路灯节能改造、城西新村路网、乾霄路改造、固体废物处置中心、畜禽无害化处置场等一批重点城建项目；推进辉乐桥、棚户区改造、金福路、市公墓园区等项目建设；开工建设金城江区第三中学新校区。加大宜州城区旧城改造力度，完善路网结构，提升美化绿化亮化水平。

稳步推进宜居城市建设。积极实施大县城战略，完善县城基础设施和公共配套，推动产业、人口向县城集聚，全面提升县域经济发展活力和综合竞争力。深入推进宜居城市创建，市中心城区宜居城市指数达到50以上，南丹、天峨达到70以上，其他县达到45以上。推动南丹撤县改市，推进宜州、环江、东兰3个县（区）新型城镇化示范县建设。全面落实农村人口市民化政策。提升城市管理水平，严厉打击“两违”行为，不断推进城市管理人性化、精细化、网格化。

（七）加快建设美丽河池，巩固提升河池生态优势。牢固树立绿水青山就是金山银山、河池生态优势金不换的理念，深入实施绿色发展战略，建设美丽河池。

加快推进生态文明示范区建设。深入推进石漠化治理，完成植树造林25万亩以上，改善生态环境。启动国家森林城市创建，抓好城市、园区、景区、道路、河流、庭院等重点区域造林绿化。全面落实自然保护区保护制度，探索推进“林长制”，加强生态公益林、自然林的保护。抓好森林公园、地质公园、湿地公园管理，加快都安澄江、东兰坡豪湖、南丹拉希3个国家湿地公园试点建设，推进红水河森林公园、凤旁林场森林公园项目申报工作。

实行最严格的生态保护制度。全面实行“河长制”，加强水资源保护、水域岸线管理、水污染防治和水环境治理，饮用水水源地水质达标率达到100%。加大工业废气、城市扬尘、机动车尾气治理，市中心城区环境空气质量优良天数达92%以上。扎实推进33个环保重点项目建设，确保环境安全。继续抓好中央环保督察反馈问题整改工作。落实最严格土地保护制度，严守耕地保护红线。

抓好宜居乡村建设。大力推进“美丽乡村”建设，巩固提升清洁乡村、生态乡村建设成果，推进宜居乡村建设，全面改善农村人居环境。继续抓好全国特色小镇宜州区刘三姐镇建设。整体推进村镇建设，基本完成26个自治区百镇示范、少数民族乡试点和54个自治区乡土特色示范点建设任务；全面完成村庄公共照明、农村改厨改厕改圈任务。大力创建文明村镇、绿色村屯和星级文明户，培育优良乡风民风。

（八）全面深化改革，提升开放合作水平。坚持把改革贯穿于经济社会发展各领域，加快推进关键性改革，主动融入区域合作，激发发展活力。

扎实推进重点领域改革。深化供给侧结构性改革，坚决落实“三去一降一补”。加大“放管服”改革力度，进一步精简行政审批事项；全面推进政务服务中心“一号一窗一网”模式；实行工业园区代办服务制度，提升政务服务水平。推进国资国企改革，基本完成国有企业“三供一业” 分离移交工作，加快剥离国有企业办社会职能；推进国有企业改制和国有“僵尸企业”处置；积极发展混合所有制经济，培育国有强优企业。推进国有林场配套改革，不断深化林权制度改革。完成排污费改环境保护税改革。统筹推进文化教育、医疗卫生、社会治理、安全生产、统计体制等领域改革，全面释放深化改革红利。

提升开放合作水平。主动融入珠江—西江经济带和北部湾经济区开放合作，积极承接产业转移，深化经贸合作。加强与“一带一路”沿线国家的合作，积极主动“走出去”。积极融入柳来河一体化，加快桂中城市群战略合作进程。加强与左右江革命老区的开放合作，积极探索与石漠化集中连片特困地区的合作与发展。面向“长三角”“珠三角”等重点区域，加大招商引资力度，着力改善营商环境，千方百计引进一批符合产业发展需要的实体经济项目，全年招商引资到位资金增长15%以上，利用外资增长15%以上。

（九）抓好财税金融工作，增强发展保障能力。坚持以服务实体经济为中心，以深化财税金融改革为主线，做大财政蛋糕，做好金融文章，防范化解金融风险，促进经济稳定增长、提质增效。

抓好财税工作。在保障基本运行、保障民生支出的基础上，把工作重点转移到服务实体经济、服务企业发展、服务项目建设上来，大力培植财源。健全预算项目储备机制，加强实体经济发展的项目谋划。强化财政政策、资金、项目的统筹，压实预算部门主体责任，加快预算执行进度，提高资金使用效益。

提高金融服务实体经济水平。围绕金融服务实体经济、防控金融风险、深化金融改革三项重点任务，推动金融业更好地回归本源、服务实体。继续实施“引金入河”，支持银行、证券、保险等金融机构到河池设立分支机构。发挥国有融资平台作用，提高融资能力，扩大融资规模。坚持政府引导、市场运作，大力推广政府和社会资本合作（PPP）机制。推动金融资源向产业发展、基础设施建设等领域聚集，力争实现新增贷款100亿元。积极发展普惠金融，优化金融生态环境，坚决守住不发生区域性、系统性金融风险的底线。

（十）保障和改善民生，营造团结和谐的社会生态。全面落实以人民为中心的发展思想，不断满足人民日益增长的美好生活需要，使人民获得感、幸福感、安全感更加充实、更有保障、更可持续。

优先发展教育。加快学前教育普及普惠，实现乡镇中心幼儿园全覆盖，新建一批村级公办幼儿园，学前三年教育毛入园率达85%。统筹推进县域义务教育城乡一体化改革发展，宜州、罗城、巴马义务教育均衡发展通过自治区督导评估验收，宜州、环江、南丹、天峨、东兰、凤山、大化通过国家评估认定。实施义务教育学校标准化建设，鼓励有条件的县（区）集中到县城办初中，义务教育巩固率达93%。提升普通高中基础能力建设，高中阶段教育毛入学率达86%。积极发展特殊教育，提高适龄残疾儿童入学率。支持和规范民办教育发展。大力发展职业教育，推进中等职业学校布局调整和专业结构优化。实施乡村教师支持计划，加强教师队伍建设。

抓好就业和社会保障工作。坚持就业优先和积极就业政策，鼓励创业带动就业，新增城镇就业1.4万人以上，新增农村劳动力转移就业5万人次。免费对贫困家庭“两后生”和贫困劳动力开展精准培训。深入推进全民参保计划，完善五项社会保险统一征缴制度。健全完善城乡居民基本医疗保险制度，做好职工医疗双保险、城乡居民大病保险、建档立卡贫困人口关爱医疗保险工作。探索实现农村低保标准与扶贫标准“两线合一”，提高城乡低保保障标准和补助水平。提升社会救助、灾害救助、社会福利、残疾人保障服务水平。新开工建设各类保障性住房1069套，基本建成1600套，完成农村危房改造1万户。

推进健康河池建设。全面实施分级诊疗，建立医疗联合体、县乡医疗卫生服务一体化体系。实施中医药壮瑶医药振兴计划。深入开展爱国卫生运动，推进河池市、巴马县和都安县创建国家卫生城市（县城），环江县、东兰县、大化县创建自治区卫生县城。加快卫生项目建设，完成市人民医院儿科中心、宜州区人民医院、南丹县工人医院、凤山县人民医院等综合医院项目和23个乡镇卫生院项目建设任务。深入实施防治艾滋病攻坚工程。完善与全面两孩政策配套的公共服务体系，加大人口出生缺陷综合防控。

推动文化繁荣兴盛。始终把意识形态工作摆在重要位置，深入开展社会主义核心价值观和中国梦宣传教育。广泛开展精神文明创建活动，深入推进社会公德、职业道德、家庭美德和个人品德建设。实施“文化惠民”“文艺精品”工程，举办第十九届铜鼓山歌艺术节。加快推进河池拔群干部学院、刘三姐博物馆建设。完善城乡文化体育基础设施，新建村级公共服务中心100个，完成农家书屋补充更新410个。

强化社会治理创新。建立健全矛盾纠纷源头治理机制，实行社会稳定风险评估，有效化解各类社会矛盾。加大全民普法力度。巩固平安建设成果，进一步加强“天网工程”建设，完善立体化社会治安防控体系，依法打击各类违法犯罪行为。建立网络综合治理体系，营造清朗的网络空间。推进群众信访服务中心标准化规范化建设。扎实开展安全生产专项整治，确保安全生产形势持续好转。切实加强食品药品安全监管，加大自治区食品安全示范城市创建力度。强化综合应急救援体系建设，提高突发公共事件应对处置能力。加强公共安全气象保障服务能力建设。加大地质灾害防治工作力度。深化全民国防教育，提升双拥共建质量，推进军民融合深度发展。认真贯彻党的民族政策，以迎接自治区成立60周年大庆为契机，深入开展民族团结进步创建活动，办好大化瑶族自治县30周年县庆。全面贯彻党的宗教工作基本方针，促进宗教关系和谐。

三、加强政府自身建设

全面贯彻党的十九大精神，以习近平新时代中国特色社会主义思想为指导，按照全面从严治党的要求从严治政，以改革创新精神加强自身建设，认真行使好人民赋予的权力，对人民负责、受人民监督、让人民满意。

（一）坚定政治立场。牢固树立“四个意识”，坚定“四个自信”， 毫不动摇地忠诚于党，毫不动摇地忠诚于人民，毫不动摇地忠诚于事业，始终在政治立场、政治方向、政治原则、政治道路上同以习近平同志为核心的党中央保持高度一致。严格遵守政治纪律和政治规矩，严格规范党内政治生活，严格落实民主集中制，营造风清气正的良好政治生态。

（二）强化理论武装。坚持用习近平新时代中国特色社会主义思想武装头脑、指导实践、推动工作。深入推进“两学一做”学习教育常态化制度化，扎实开展“不忘初心、牢记使命”主题教育，深刻领会和掌握十九大报告提出的一系列新的重要观点、重大论断、重大举措，把学习成果转化为促进发展的具体措施和务实成效。

（三）严格依法行政。深化法治政府建设，完善重大行政决策专家咨询论证、公众参与决策等制度。加强行政复议和仲裁工作，依法有效化解矛盾纠纷。完善政府立法工作机制。自觉接受人大及其常委会的法律监督和工作监督，自觉接受人民政协的民主监督，高质量做好建议、提案办理。认真听取民主党派、工商联和人民团体的意见建议，自觉接受社会舆论监督。

（四）提升执行力。继续解放思想，强化责任担当，把该负的责任负起来，把该做的工作做到位。坚持言必行、行必果，认准的事情一抓到底，承诺的事项坚决兑现，做到雷厉风行、快捷高效。

（五）加强廉政建设。严格落实全面从严治党主体责任，全面落实党风廉政建设责任制，认真履行“一岗双责”，加大反腐倡廉力度。坚决贯彻落实中央八项规定精神，驰而不息整治“四风”。深化政务公开和政府信息公开，坚持以公开为常态，不公开为例外，依法依规、公平公正、公开透明抓好项目建设。加强行政监察和审计监督，开展扶贫领域腐败和作风问题专项治理，强化监督执纪问责，坚决纠正损害群众利益的不正之风。

各位代表！河池前景无限美好，赶超跨越重任在肩。我们要紧密团结在以习近平同志为核心的党中央周围，在自治区党委、政府和市委的坚强领导下，全面贯彻党的十九大精神，以习近平新时代中国特色社会主义思想为指导，不忘初心、牢记使命，开拓进取、攻坚克难，推动全市经济社会持续健康发展，为坚决打赢脱贫攻坚战、同步全面建成小康社会而努力奋斗！
